# P040. Primary Valsalva maneuver headache without primary cough headache

**DOI:** 10.1186/1129-2377-16-S1-A130

**Published:** 2015-09-28

**Authors:** Enrico Ferrante

**Affiliations:** Headache Centre, Neurosciences Department Niguarda Cà Granda Hospital, Milan, Italy

## Background

The uncommon headache related to a Valsalva maneuver (VM) is coded within the International Headache Society Classification (ICHD-3beta) among “Other primary headaches”, 4.1 “Primary cough headache” (PCH). This chapter deals with headaches precipitated by coughing or straining in the absence of any systemic or intracranial disorder. The clinical features of the pain are characterized by sudden onset, lasting from 1 s to 2 hours, and brought on by and occurring only in association with coughing, straining, and/or a VM. Neuroimaging plays an important role in differentiating secondary forms.

## Case report

A 71-year-old woman with obesity, high blood pressure, type 2 diabetes, hypothyroidism and glaucoma was admitted because for the past 18 months she had headache attacks with stabbing severe pain (NVR 10/10), on the right fronto-temporal side with parietal-occipital diffusion. The pain lasted from 30 to 360 minutes with and without intake of analgesic, respectively. For the first 6 months, the headache was triggered only when bending over. Subsequently, also other activities that required the execution of a Valsalva maneuver (coughing, sneezing, laughing) caused the same headache attacks. Neurological examination and hematologic work up were normal. MRI of the brain, angiography and CSF circulation study were normal. The patient was treated with indomethacin 50 mg daily for 4 days and then 50 mg twice a day for two months. The headache gradually disappeared after 5 days of therapy. After 12 months of follow-up the patient is in good health.

## Discussion and conclusions

The pathophysiology of headache triggered by VM is unknown. The most reasonable hypothesis is a sudden increase in intracranial pressure secondary to high venous pressure, such as during coughing; other reported contributing factors are transient post-infective hypersensibility of pressure receptors localized in venous vessels, reduction of the posterior fossa volume with consequent crowding of its structures, incompetence of the valves of the internal jugular veins, venous stenosis, and intermittent intraocular pressure elevation. The initial history of our case and some cases reported in the literature suggest that the headache caused by maneuvers related to increased intrathoracic pressure (Valsalva's maneuver like stimuli) may not be triggered by coughing. In these cases, when structural lesions are excluded by MRI or similar tests, we propose the eponym, “primary Valsalva maneuver headache” (which is more appropriate than the equivalent [“PCH”]). In these patients it is possible that the Valsalva maneuver provoked by the cough did not reach the pain threshold to cause headache.

Written informed consent to publication was obtained from the patient(s).

